# Regulatory T cells and control of the germinal centre response

**DOI:** 10.1186/s13075-014-0471-7

**Published:** 2014-10-30

**Authors:** Ine Vanderleyden, Michelle A Linterman, Kenneth GC Smith

**Affiliations:** Lymphocyte Signalling and Development, Babraham Institute, Babraham Research Campus, Cambridge, CB22 3AT UK; IB Autoimmune Genetics Laboratory, KU Leuven, O&N 2, Campus Gasthiusberg, Herestraat 49, bus 1026, 3000 Leuven, Belgium; Cambridge Institute for Medical Research, Wellcome Trust/MRC Building, Cambridge Biomedical Campus, Cambridge, CB2 0XY UK

## Abstract

Germinal centres (GCs) are specialised lymphoid microenvironments that form in secondary B-cell follicles upon exposure to T-dependent antigens. In the GC, clonal expansion, selection and differentiation of GC B cells result in the production of high-affinity plasma cells and memory B cells that provide protection against subsequent infection. The GC is carefully regulated to fulfil its critical role in defence against infection and to ensure that immunological tolerance is not broken in the process. The GC response can be controlled by a number of mechanisms, one of which is by forkhead box p3 expressing regulatory T (Treg) cells, a suppressive population of CD4^+^ T cells. A specialised subset of Treg cells – follicular regulatory T (Tfr) cells – form after immunisation and are able to access the GC, where they control the size and output of the response. Our knowledge of Treg cell control of the GC is expanding. In this review we will discuss recent advances in the field, with a particular emphasis on the differentiation and function of Tfr cells in the GC.

## Introduction

The establishment of antigen-specific memory responses is a key aspect of adaptive immunity that protects the host against future infections and forms the basis of successful immunisation. Germinal centres (GCs) are specialised microenvironments that form in B-cell follicles within secondary lymphoid organs upon infection or immunisation with a T-dependent antigen. The effector products of the GC responses are long-lived, high-affinity antibody secreting cells and memory B cells [1].

The GC response is initiated when B cells encounter antigen within the secondary lymphoid tissues. Naïve B cells recirculate through secondary lymphoid tissues and enter the B-cell follicle, located underneath the subcapsular sinus in the lymph nodes and underneath the marginal zone in the spleen, near sites of antigen entry [2]. In the follicle, naïve B cells scan for their specific antigen and are activated following engagement of their B-cell receptor (BCR) by small soluble antigens directly, by antigen presentation from subcapsular sinus macrophages [3-5], or by taking up antigen from follicular dendritic cells (FDC) [6]. After antigen encounter, B cells rapidly upregulate C-C chemokine receptor type 7 (CCR7), whose ligands chemokine (C-C motif) ligand (CCL)21 and CCL19 are expressed in the adjacent T-cell zone. B cells use this gradient to migrate towards the T:B border, where they engage in cognate interactions with CD4^+^ T-helper type (Th) cells [7]. B cells then upregulate the orphan G protein-coupled receptor Epstein–Barr virus-induced gene 2 (EBI2), allowing the B cell to migrate to the outer edges of the follicle [8,9]. After division, B cells either take part in the extrafollicular antibody response or enter the B-cell follicle to seed the GC [10].

B cells that differentiate into extrafollicular plasma cells secrete class-switched or non-class-switched antibodies in the early phase of infection and undergo apoptosis *in situ* after a few days [11]. This initial and rapid burst of antibody production is an important component of the early immune response against infectious organisms and allows time for the GC to mature without compromising host defence during this time [12].

B cells that enter the B-cell follicle to seed the GC begin to divide rapidly, and after this initial clonal expansion the GC divides into two distinct zones: the dark zone and the light zone. In the dark zone, B-cell clones undergo somatic hypermutation, which introduces random point mutations in the V regions of their immunoglobulin genes [13]. This process is followed by affinity-based selection in the light zone that contains FDC bearing immune complexes and follicular helper T (Tfh) cells. B cells with somatically mutated BCRs collect antigen from the surface of FDC, internalise it and present it to Tfh cells in the context of major histocompatibility complex class II (MHC-II). B cells with the highest affinity BCRs are able to outcompete lower affinity B cells for T-cell help, resulting in further clonal expansion of high-affinity GC B cells and formation of high-affinity plasma cells and memory B cells [14,15]. This process of mutation and selection that generates effector B cells with BCRs with increased affinity for antigen is referred to as affinity maturation, and competition for Tfh cell help is an essential mediator of this [15].

### Follicular helper T cells

Tfh cells are essential for the formation and maintenance of the GC response [16]. Tfh differentiation is initiated by priming of the CD4^+^ T cell by dendritic cells (DCs) via the engagement of the T-cell receptor (TCR) by the MHC-II peptide complex on DCs in conjunction with co-stimulation between CD80/CD86 on the DC and CD28 on the T cell. During these T:DC interactions, the cytokines IL-6 and IL-12 and the co-stimulatory molecule inducible co-stimulator (ICOS) support differentiation into Tfh precursor cells [17]. These signals are critical for induction of the transcription factor B-cell lymphoma (Bcl)-6 [18], which is both necessary and sufficient for Tfh differentiation [19-21]. Bcl-6 promotes Tfh differentiation by actively repressing the Th1 (Tbet), Th2 (GATA-binding-protein 3 (GATA3)), Th17 (retinoid-orphan receptor gamma (RORγt)) and regulatory T (Treg) (forkhead box p3 (Foxp3)) master transcription factors as well as the transcription factor B-lymphocyte-induced maturation protein 1 (Blimp-1) [19-21]. Bcl-6 and Blimp-1 are mutually antagonistic and the balance of these two transcription factors is essential for optimal Tfh cell differentiation [21].

Expression of CXCR5 allows T cells to migrate to the T:B border towards the ligand for CXCR5, CXCL13, which is expressed by FDC in the follicle [22,23]. Induction of CXCR5 on Tfh precursor cells is mediated by the expression of the transcription factor achaete-scute complex homolog 2 (Ascl-2) [24]. After T-cell priming, Tfh precursor cells need to interact with B cells in order to fully differentiate into GC Tfh cells; these stable interactions between Tfh precursor cells and B cells are mediated by ligation of the SLAM family of receptors, CD84 and Ly108, supported by the intracellular adaptor molecule SLAM-associated protein (SAP) [25]. Tfh cells also receive signals through ICOS during interactions with bystander B cells; this induces the phosphatidylinositide 3-kinase pathway and triggers actin waves and polarised pseudopod formation, facilitating the migration of Tfh cells to the follicle [26]. Once within the GC, Tfh cells provide help to GC B cells.

Fully differentiated GC Tfh cells provide survival and differentiation signals to GC B cells through CD40 ligand–CD40 interactions and secretion of cytokines, including IL-21 and IL-4 [27-31]. GC B cells compete with each other for help from Tfh cells by presenting antigen on MHC-II. Those GC B cells that can engage Tfh cells will receive survival and differentiation cues to exit the GC as long-lived plasma cells or memory cells or to undergo further rounds of somatic hypermutation. The B cells with the highest affinity BCRs are able to collect and then present the most antigen to Tfh cells, thereby outcompeting the lower affinity B cells for T-cell help. The dialogue between Tfh and GC B cells is not one directional, with B cells providing signals to Tfh cells that control their maintenance and function. PD-1 ligation on Tfh cells during interaction with B cells controls the Tfh cell number, and secretion of the cytokine IL-21 and CD80 expression on B cells supports Tfh survival [32,33]. Together, the priming of naïve CD4^+^ T cells leads to specific changes in gene transcription that culminates in Tfh differentiation and migration into the GC, where they provide essential help to GC B cells for their selection, survival and differentiation.

## Control of the germinal centre by Foxp3^+^ regulatory T cells

### Foxp3^+^ regulatory T cells

The size and specificity of the GC is influenced by a number of factors such as antibody feedback [34], Qa-1^+^CD8^+^ Treg cells [35] and Foxp3^+^ Treg cells. This review will focus on the latter and their role in controlling the GC response. Foxp3^+^ Treg cells are a subset of CD4^+^ T cells that suppress immune responses and are essential for maintaining immunological tolerance [36]. Treg cells can develop in the thymus and these Foxp3^+^ cells are referred to as thymic Treg cells [37,38]. In addition to thymic Treg cells, recent thymic emigrants can switch on Foxp3 expression and become suppressive Treg cells [39]. Treg cells originating from the thymus have a TCR repertoire whose specificity is skewed towards self-antigen and is distinct from that of conventional effector T cells [40]. Treg cells can also differentiate from non-Treg CD4^+^ T cells that switch on Foxp3 expression in the periphery; these are referred to as peripheral Treg cells [41]. Finally, Treg cells can be generated *in vitro* in the presence of transforming growth factor beta and are referred to as induced Treg cells [38].

The transcription factor Foxp3 is essential for the suppressive function of Treg cells. In humans, mutations in *FOXP3* lead to immune dysregulation, polyendocrinopathy, enteropathy, and X-linked syndrome (IPEX), which is characterised by severe systemic autoimmunity. Similarly, deficiency of Foxp3 in mice (scurfy mice) leads to unrestrained T-cell and humoral immune responses, which include spontaneous GC formation and Tfh cell expansion in the absence of immunisation or infection [42-44]. Mice in which Foxp3^+^ cells lack interferon regulatory factor (IRF)4 develop spontaneous GCs but are not a phenocopy of Foxp3-deficient mice [45]. This observation suggests that IRF4 controls an aspect of Treg biology that is essential for moderating the GC itself and that specific, cell-intrinsic molecular pathways mediate the suppression of the spontaneous GC response by Treg cells. IRF4 is required for expression of the homing molecules CD62L and CD103 and the inhibitory molecule cytotoxic T-lymphocyte antigen 4 (CTLA-4), and changes in these molecules may have implications for Treg control of the GC. There is evidence that CTLA-4 is also a critical regulator of the GC as CTLA-4-deficient mice develop spontaneous GCs [46]. CTLA-4 is expressed at high levels on Treg cells and exerts its inhibitory function by depleting CD80/CD86 from the surface of antigen-presenting cells by trans-endocytosis, thereby inhibiting co-stimulatory signalling [47]. This inhibition results in failure of DCs to activate T cells through CD28, thereby inhibiting priming of CD4^+^ T cells. Taken together, these findings demonstrate that Foxp3^+^ Treg cells play a role in suppressing the initiation of the GC, and CTLA-4 is a probable effector mechanism by which Treg cells may mediate this suppression. It could be speculated that extrafollicular Treg cells suppress GC initiation by means of CTLA-4-mediated inhibition of T-cell proliferation and subsequent Tfh cell differentiation.

In contrast to the finding that Foxp3^+^ Treg cells suppress the GC response, Leon and colleagues demonstrated that Treg cells can promote GC responses [48]. Using influenza infection in a strain of mice where treatment with diphtheria toxin specifically eliminates all Foxp3^+^ cells, they demonstrated that ablation of Treg cells results in a decrease in antigen-specific GC B cells [48]. In this model, Treg suppression of the GC was mediated by limiting the availability of IL-2. IL-2 signalling negatively impacts on Tfh differentiation by upregulating the transcription factor Blimp-1 in a signal transducer and activator of transcription (STAT)5-dependent manner [49,50], suggesting that when Treg cells are not able to limit IL-2, primed T cells are skewed away from the Tfh lineage due to increased IL-2 signalling. These data seem to be in contrast to the data described above that show that Foxp3^+^ Treg cells inhibit the GC response. However, work from Wing and Sakaguchi demonstrated that Treg cell control of the GC differs depending on the time point of the response in which they are acting. Depletion of Treg cells at the time of immunisation enhances the number of antigen-specific Tfh cells and GC B cells, whereas depletion later in the response leads to expansion of non antigen-specific GC B cells and Tfh cells, at the expense of cells specific for the responding antigen [46]. Consistent with this, Leon and colleagues depleted Treg cells on days 0, 4 and 7 relative to influenza infection followed by analysis on day 10 [48]. These time points are prior to the peak of the GC response following influenza infection [51], a time point during which Treg cells promote the initiation of the immune response by supporting effector CD4^+^ T-cell differentiation rather than suppressing the GC response.

**Figure 1:**
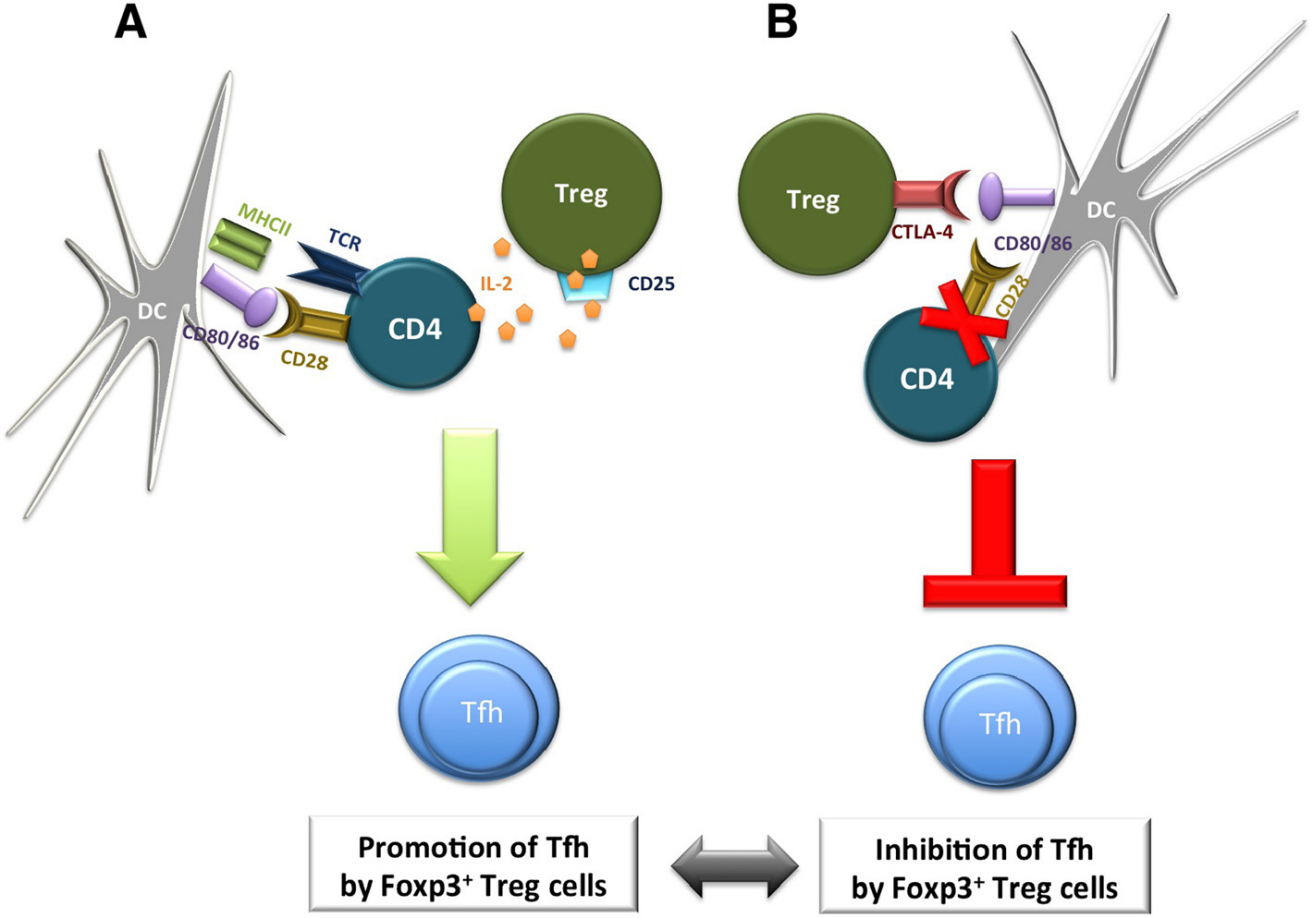
**Extrafollicular Foxp3** ^**+**^ **regulatory T cells control the initiation of the germinal centre response. (A)** During the early germinal centre (GC) response, Foxp3^+^ regulatory T (Treg) cells promote the immune response by IL-2 consumption. **(B)** A model of Foxp3^+^ Treg cell-mediated GC suppression by cytotoxic T-lymphocyte antigen 4 (CTLA-4). CTLA-4 is able to trans-endocytose CD80/86 from the dendritic cell (DC), thereby inhibiting co-stimulatory signalling through CD28 on the CD4^+^ T cells. Defective T-cell priming will result in failure of follicular helper T (Tfh) cell differentiation. Foxp3, forkhead box p3; MHC-II, major histocompatibility complex class II; TCR, T-cell receptor.

These data also suggest that there might be an important difference between Treg cells controlling the GC from outside the follicle by controlling its initiation and follicular regulatory T (Tfr) cells controlling the GC from inside where they regulate the size and output of the response. Taken together, extrafollicular Treg cells may inhibit the formation of a GC response in the absence of immunisation or infection via CTLA-4 trans-endocytosis. But these cells may also enhance the initiation of the GC response after infection by limiting IL-2 availability and thereby promoting Tfh differentiation (Figure 1).

### Follicular regulatory T cells

Tfr cells are a subset of Foxp3^+^ Treg cells that are found within the GC, where they act to control the magnitude and output of the GC response. Lim and colleagues first identified Foxp3^+^ cells within human tonsil GCs in 2004 [52]. These authors demonstrated that addition of CD4^+^CD25^+^CD69^−^ T cells to co-cultures of CD57^+^ Th cells with naïve, GC or memory B cells isolated from tonsils suppressed antibody production. The CD4^+^CD25^+^CD69^−^ T-cell populations were shown to contain *FOXP3*-expressing cells. Although the authors suggest that some of these cells can be identified in the GC, these CD4^+^CD25^+^CD69^−^ Treg cells highly express CCR7, whose ligands are not expressed in the GC, and not CXCR5, the key migration cue for access to the GC. Therefore, it is unlikely that these CD4^+^CD25^+^CD69^−^ Treg cells are all located within the GC but they may control the response from outside the follicle.

Interestingly, activation of CD4^+^CD25^+^CD69^−^ Treg cells increased the expression of CXCR5, facilitating their migration towards CXCL13. This indicates that TCR activation is able to change the chemotactic profile of Treg cells to facilitate migration into the GC. The CD69^+^ counterparts of these Treg cells do express CXCR5, and it could be speculated that upon activation CD4^+^CD25^+^CD69^−^ Treg cells start to express CD69 and CXCR5 to migrate to the GC. Following on from this work, in 2011 three independent groups demonstrated that Foxp3^+^CD4^+^ T cells are able to undergo functional differentiation into a specialised subset of Treg cells that migrate into the GC and control its size and output directly [44,53,54]. These Treg cells located inside the GC are now referred to as Tfr cells.

#### The origin of follicular regulatory T cells

Tfr cells are a distinct T-cell population within the GC that phenotypically mirror Tfh cells in many aspects, including the expression of several Tfh cell markers such as Tfh lineage-specific transcription factor Bcl-6 along with CXCR5, PD-1, ICOS, and SAP [44,53]. Tfr cells do not express the B-cell helper molecules IL-21, IL-4 and CD40 ligand that are characteristic of Tfh cells [53]. Instead they express a range of typical Treg cell markers in addition to Foxp3, such as glucocorticoid-induced tumour necrosis factor receptor-related protein and CTLA-4 [44,53]. Gene expression profile analysis further showed that Tfr cells have a distinct transcriptional profile that has more in common with Treg cells than Tfh or other Th cells [53].

Because Tfr cells have phenotypic characteristics of both Tfh and Treg cells, it seemed likely that these cells arise from one or the other of these cell types. Thus, Tfr cells could derive from Foxp3^+^ Treg cells that enter the GC or from Tfh cells that switch on Foxp3 within the GC. To determine the origin of Tfr cells, naïve Foxp3^−^ TCR-transgenic T cells were transferred into intact recipient mice prior to immunisation. Tfr cells develop exclusively from recipient cells, whereas Tfh cells developed from the TCR-transgenic T cells, suggesting Tfr cells do not derive from Tfh cells [53]. Supporting this, adoptive transfer of Foxp3^+^CD4^+^ or Foxp3^−^CD4^+^ cells into recipient mice showed that Tfr cells derive from Treg cells and not Foxp3^−^ precursors [44,53] and Tfr cells do not develop in mice depleted of Treg cells at the time of immunisation [53]. Moreover, Tfh cells in culture are resistant to conversion into Foxp3^+^ cells under Treg cell polarising conditions [54]. Together, these data indicate that Tfr cells derive from Treg cells rather than from Tfh cells.

#### Differentiation and maintenance of follicular regulatory T cells

Initial experiments using mixed bone marrow chimaeras showed that Tfr cells require a number of differentiation cues used in Tfh cell development [53]. Tfr cell development requires CD28 and ICOS ligation as well as the presence of B cells and SAP expression, which facilitates T:B interactions [53,55]. Like Tfh cells, Tfr cells absolutely require Bcl-6 expression, suggesting that common developmental cues may be responsible for inducing their shared transcriptional regulator [44]. In contrast to Tfh cells, in which Bcl-6 can repress Foxp3 expression, Tfr cells co-express Bcl-6 and Foxp3. However, the mechanism by which these transcription factors can be expressed within the same cell type, and the implications of this for Tfr cell biology, remain to be demonstrated. Although *Bcl6*^*−/−*^ mice are unable to form Tfr cells [44], Bcl-6 is not required for the initial upregulation of CXCR5. In Tfr cells, CXCR5 upregulation depends on the transcription factor NFAT2 [56]. Strikingly, NFAT2 deficiency in T cells impaired CXCR5 expression by Foxp3^+^CD4^+^ cells and resulted in an enhanced GC response. This effect was specific to Tfr cells and Tfh cell migration was not affected by NFAT2 deficiency. NFAT2 is thus essential to initiate GC homing in Tfr cells, playing an analogous role to Ascl-2 in Tfh cells. This suggests that Bcl-6 might be required for stabilising or maintaining Tfr cell development rather than inducing it.

These data also indicate that, despite their similar phenotype, Tfh cells and Tfr cells have distinct molecular regulation of CXCR5 expression and therefore these cells do not entirely share the same molecular differentiation pathway. Other molecules have been implicated in Tfr differentiation, such as tumour necrosis factor receptor-associated factor 3 (TRAF3) [57]. Ablation of TRAF3 in Foxp3^+^ Treg cells results in a decrease in the number of Tfr cells, accompanied by a larger GC response and increased antibody production [57]. In contrast, expression of the helix–loop–helix proteins Id2 and Id3 in Treg cells represses Tfr cell development, as mice deficient in these molecules have spontaneous Tfr cell formation [58]. Together, these data demonstrate that Tfr cells require specific cues for their formation from Foxp3^+^ CD4^+^ T cells, and if their development is impaired the GC is larger.

In addition to particular cues being required for their development, the size of the Tfr cell population is also controlled at a cell-intrinsic molecular level. In contrast to Tfh cells, a proportion of Tfr cells express Blimp-1, a transcription factor important in Treg cell function and for repressing Tfh cell development [59]. In the absence of Blimp-1, the Tfr cell population doubles in size, indicating that Bcl-6 and Blimp-1 have reciprocal effects on Tfr cell numbers, enhancing and reducing the size of the population, respectively [53]. Tfr cells also have high expression of the receptor PD-1, an inhibitory molecule that can dampen lymphocyte responsiveness to antigen [53,55]. Adoptive transfer experiments using cells from PD-1-deficient mice showed that PD-1 expression limits the size of the Tfr cell population [55]. Together, these experiments demonstrate that Blimp-1 and PD-1 limit the size of the Tfr cell pool. Tight control of Tfr cell numbers is likely to be required to ensure appropriate, and avoid excessive, suppression of the GC.

#### Follicular regulatory T-cell function

**Figure 2:**
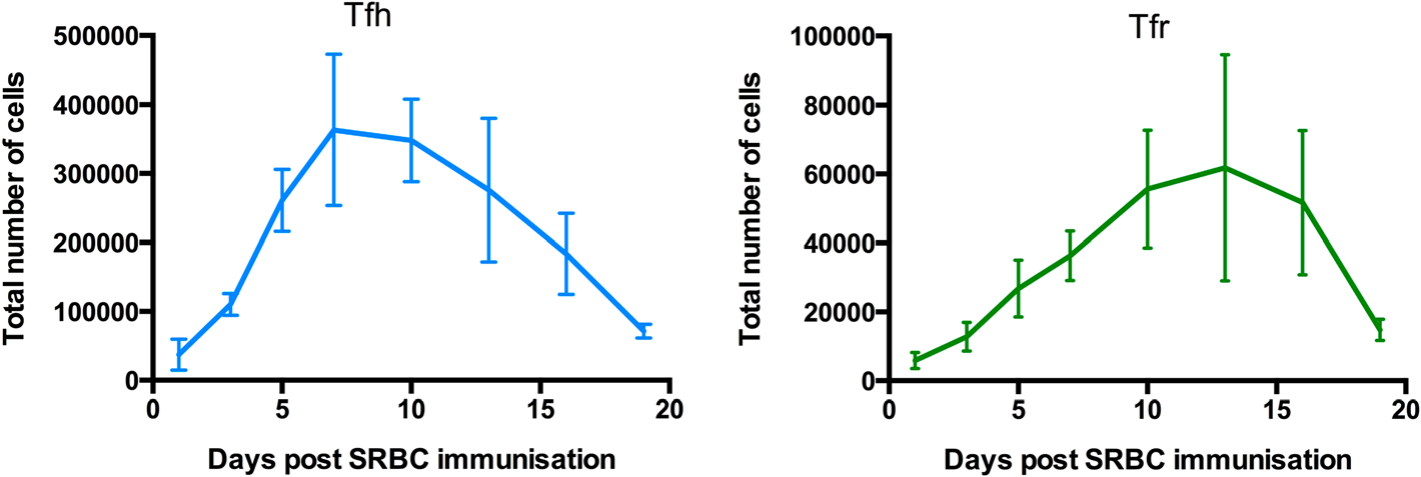
**Follicular helper T cells and follicular regulatory T cells have different kinetic profiles.** C57BL/6 mice were immunised intraperitoneally with sheep red blood cells (SRBC) and the numbers of splenic follicular helper T (Tfh) cells and follicular regulatory T (Tfr) cells were assessed by flow cytometry at different time points. During the germinal centre response, Tfh cells peak slightly earlier compared with Tfr cells. Tfh cell numbers peak around days 7 to 11 whereas the highest number of Tfr cells can be observed from days 11 to 17.

It is tempting to speculate that the absence of Tfr cells might account for the spontaneous formation of GCs observed in Foxp3-deficient mice. Consideration of the kinetics of Tfr and Tfh cell formation argues against such a role for Tfr cells in controlling the initiation of the GC. Tfr cells are barely detectable in unimmunised mice and, after sheep red blood cell immunisation, Tfh cell numbers peak at days 7 to 11, prior to Tfr cells (days 11 to 17) (Figure 2). This difference demonstrates that Tfr cells are generated in response to immunisation, not prior to GC induction, and are therefore unlikely to suppress the formation of the GC response. The kinetics of Tfr cell formation suggest that Tfr cells are more likely to control the function and output of an established GC, with extrafollicular Treg cells controlling the initiation of the GC, as discussed earlier in this review.

*In vitro* Treg cell suppression assays showed that Tfr cells have a similar suppressive capacity compared with Treg cells [53]. However, their role *in vivo* has not been as easy to dissect. The three initial groups describing Tfr cells in mice used different experimental strategies to determine their function *in vivo*, leading to conflicting results. In our study, mixed bone marrow chimaeras (SAP-deficient mice and Foxp3-DTR mice) were used to examine the effect of a specific reduction in Tfr cells on the GC response*.* In this system, immunisation with a T-dependent antigen significantly increased the number of Tfh cells and GC B cells, indicating that Tfr cells can suppress features of the GC response *in vivo*. When the number of Tfr cells was reduced, a slight decrease in high-affinity antibodies was also observed. In addition, antigen-specific GC B cells were fewer in number, suggesting that Tfr cells can partly control the GC response by limiting development of non antigen-specific B cells [53]. In contrast, Chung and colleagues used an adoptive transfer model in which *Cxcr5*^*−/−*^ or *Bcl6*^*−/−*^ Treg cells together with naïve CD4^+^ T cells were transferred into *Tcrb*^−/−^ mice. An increase in the number of GC B cells as well as antibody titres of both high and low affinity could be observed after immunisation, although the number of Tfh cells was unaffected [44]. Similar results were obtained in the study by Wollenberg and colleagues, in which transfer of *Cxcr5*^*−/−*^ Treg cells into *Tcra*^*−/−*^ mice resulted in an increased GC response characterised by increased antibody secretion [54]. A later study demonstrated that Tfr cells could suppress both activation and proliferation of responder T cells and IgG production *in vitro* [55].

While these studies agree that Tfr cells can limit the size of the GC response, whether they also control antigen-specific antibody production remains contentious. The discrepancies between these studies may arise from the use of different immunisation and experimental protocols undertaken in slightly different environmental conditions. An additional possible explanation might be the fact that knocking-out either CXCR5, SAP or Bcl-6 in Treg cells in these experiments might not affect the Tfr cell population alone, but may also have an impact on other aspects of Treg cell biology. New tools will need to be generated to fully understand the role that Tfr cells play in the GC.

#### Direct or indirect suppression by follicular regulatory T cells

While it is clear that Tfr cells act to control the size of the GC response, the mechanism by which they do this has yet to be defined. They could act directly on B cells or Tfh cells through cell–cell interaction, via cytokine production, or through a combination of these. Treg cells can directly inhibit B-cell antibody production *in vitro* in a cell contact-dependent manner [60]. Tfr cells cannot form in the absence of B cells or SAP, suggesting that Tfr cells or Tfr precursor cells interact with B cells *in vivo* [53]. Thus, Tfr cells may limit the GC response by direct interaction with GC B cells. However, Treg cells are also able to suppress effector T cells directly *in vitro* [52], so Tfr cells may also act by suppressing the expansion or function of Tfh cells through direct contact. One key molecule highly expressed by Treg cells that is implicated in suppression of GC responses is CTLA-4 [61]. When CTLA-4 signalling is blocked during an established GC response, the GC B cells continue to proliferate and GC resolution is prevented. Furthermore, the direct suppression of B cells *in vitro* by Treg cells is partially mediated by CTLA-4 [60]. Thus CTLA-4, in addition to its role in suppressing the initiation of the GC response, may also help in mediating Tfr cell suppression of the GC response.

**Figure 3:**
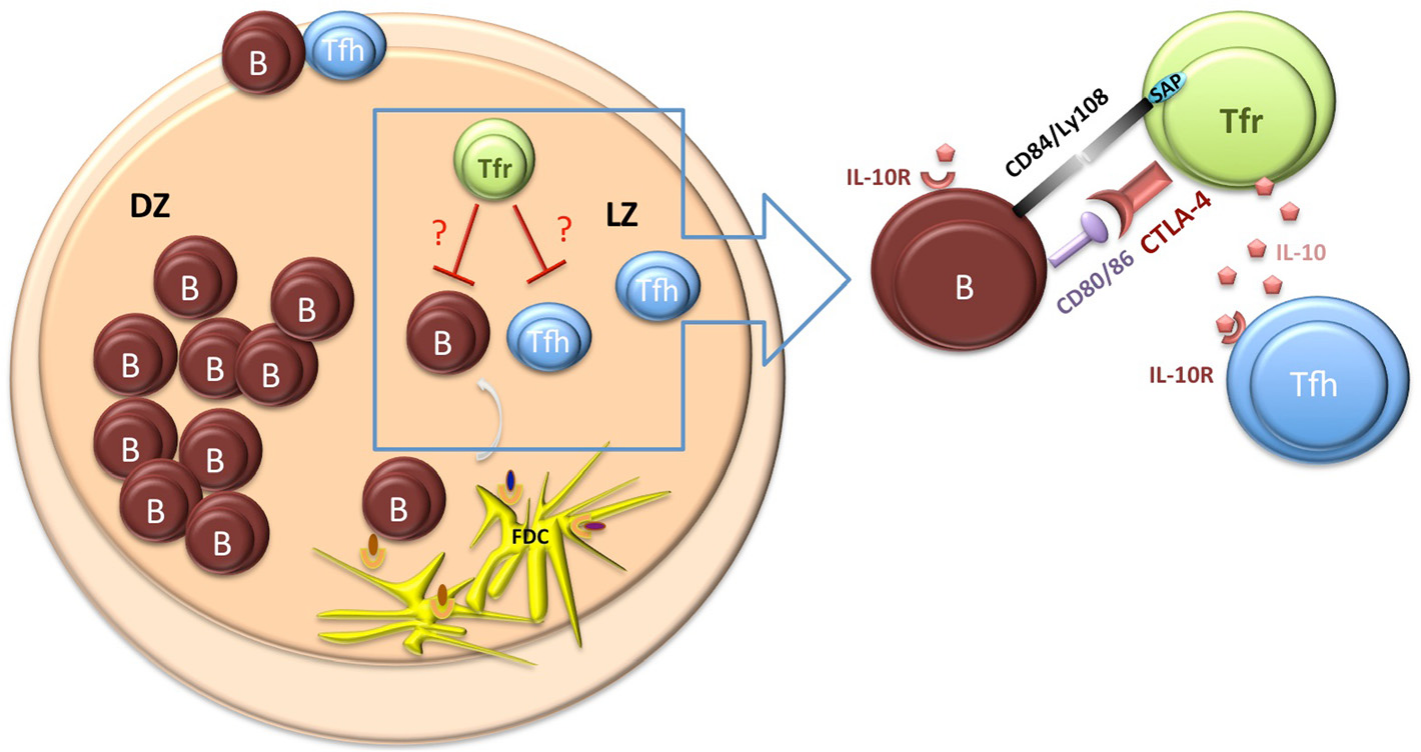
**Follicular regulatory T cells control the output of the germinal centre response.** Direct suppression of germinal centre B cells might occur through cytotoxic T-lymphocyte antigen 4 (CTLA-4)-mediated inhibition of CD80/CD86 co-stimulatory signalling. Alternatively, follicular regulatory T (Tfr) cell-mediated suppression may take place in an indirect manner by means of IL-10 secretion acting on follicular helper T (Tfh) cells. DZ, dark zone; FDC, follicular dendritic cells; LZ, light zone.

Tfr cells may also exert their suppressive effects in the GC indirectly via cytokine secretion. One candidate would be IL-10, which has an established role in mediating Treg suppression and is expressed by Tfr cells [16]. Mice with Foxp3^+^ Treg cells deficient in IL-10 do not develop severe autoimmunity but do develop spontaneous gastrointestinal inflammation [62]. The role of IL-10 during immune responses is complex and both IL-10 and its receptor (IL-10R) are broadly expressed by different immune cell types [63]. IL-10 limits the size of the population of CD4^+^CXCR5^+^ T cells in secondary lymphoid organs [64] and seems to be involved in a positive feedback loop, enhancing its own production by Treg cells [65]. Because IL-10 deficiency in Foxp3^+^ Treg cells does not result in excessive autoantibody formation, it seems likely that IL-10 production by Tfr cells may play a minor role in controlling Tfh cells rather than mediating major suppressive effects. Tfr cells are thus likely to exert their suppressive effect on distinct cell types in the GC by a number of mechanisms (Figure 3).

#### Follicular regulatory T cells and their potential role in autoimmunity

Somatic hypermutation of B-cell immunoglobulin genes within the GC can potentially generate autoreactive B-cell clones due to the random nature of the induced point mutations. Elimination of these self-reactive clones from the GC is therefore important for maintaining immunological tolerance and preventing autoimmunity [66]. Tfh cells are an important peripheral tolerance checkpoint within the GC to ensure that B cells with self-reactive BCRs do not exit the GC as long lived-plasma cells and memory B cells. *Sanroque* mice have aberrant accumulation of Tfh cells that drive spontaneous GCs and autoimmunity, indicating that Tfh cells can support self-reactive GCs [67,68].

It is also possible that Tfr cells may contribute to maintaining GC tolerance as they have been reported to limit the number of non antigen-specific GC B cells [53] and they derive from Treg precursors that possess TCRs skewed towards self-antigens. In support of this hypothesis, intravenous Ig treatment in collagen-induced arthritis in mice has been shown to reduce pathology and is associated with increased numbers of Tfr cells and a decrease in the number of GC B cells [69]. In addition, the importance of Tfr cells in autoimmune diseases has recently also been suggested in autoimmune BXD2 mice that are characterised by spontaneous autoreactive GC formation [70]. In BXD2 mice, IL-21 promotes GC formation by selective expansion of Tfh cells skewing the Tfh/Tfr ratio towards Tfh cells. Lack of IL-21 in BXD2 mice results in increased numbers of Tfr cells, and, importantly, transfer of these Tfr cells can decrease GC formation and autoantibody production in BXD2 mice [70]. Together, these data suggest that skewing the Tfh/Tfr balance potentially contributes to autoimmune pathology, and correction of this balance could have therapeutic potential.

#### Follicular regulatory T cells as a specialised regulatory T-cell subset

In recent years it has become increasingly clear that, like CD4^+^ Th cells, Treg cells are functionally and phenotypically diverse. Foxp3^+^ Treg cells can differentiate into several functional states characterised by expression of specific transcription factors classically associated with different Th-cell subsets. For example, during a Th1-skewed response, Foxp3^+^ Treg cells upregulate Tbet in a STAT1-dependent manner, the key transcription factor for Th1 cell differentiation [71,72]. Tbet expression in Treg cells drives CXCR3 expression, as it does in Th1 cells, and enables these Th1-like Treg cells to migrate towards the site of inflammation where they can specifically regulate Th1 responses. The induction of these CXCR3^+^Tbet^+^ Treg cells occurs in an interferon gamma-dependent manner, similar to Th1 cells [72]. Th17 cell-mediated immune responses can be specifically controlled by Treg cells expressing Th17-associated STAT3, as Treg cell-specific deletion of STAT3 results in fatal intestinal inflammation and excessive Th17 responses [73]. Tfh cells are the critical Th cell population for the GC, and Tfr cells phenotypically resemble Tfh cells in many respects, as described in this review. Tfr cells thus fit in a model in which Treg cells co-opt aspects of specific Th cell differentiation pathways, enabling them to migrate to sites where these Th cells are functioning in immune responses. Once there, their suppressive effects help ensure that collateral damage is minimised during the fight against infection.

## Conclusions

Tfr cells are a subset of Foxp3^+^ Treg cells that adopt features of Tfh cells to enable them to migrate into the GC while maintaining their suppressive function. These Tfr cells control the magnitude of the GC response and form within the GC, whereas extrafollicular Foxp3^+^ Treg cells likely regulate the initiation of, and input to, the GC response. Although it is clear that Tfr cells exert a suppressive function in the GC, some controversy remains about mechanisms and targets of their suppression. Further research needs to be done in order to unravel the exact mechanisms by which Tfr cells suppress the GC response, to disentangle the intrafollicular and extrafollicular roles of Foxp3^+^ Treg cells and to determine to what extent Tfr cells play a role in the prevention of GC-derived autoimmunity.

## Abbreviations

Bcl-6: B-cell lymphoma 6
BCR: B-cell receptor
Blimp-1: B-lymphocyte-induced maturation protein 1
CCL: chemokine C-C ligand
CCR: chemokine C-C receptor
CTLA-4: cytotoxic T-lymphocyte antigen 4
DC: dendritic cell
FDC: follicular dendritic cells
Foxp3: forkhead box p3
GC: germinal centre
ICOS: inducible co-stimulator
Ig: immunoglobulin
IL: interleukin
IRF: interferon regulatory factor
MHC-II: major histocompatibility complex class II
SAP: SLAM-associated protein
STAT: signal transducer and activator of transcription
TCR: T-cell receptor
Tfh: follicular helper T
Tfr: follicular regulatory T
Th: T-helper type
TRAF3: tumour necrosis factor receptor-associated factor 3
Treg: regulatory T
